# Phenylacetonitrile from the Giant Knotweed, *Fallopia sachalinensis*, Infested by the Japanese Beetle, *Popillia japonica*, Is Induced by Exogenous Methyl Jasmonate

**DOI:** 10.3390/molecules16086481

**Published:** 2011-08-03

**Authors:** Koji Noge, Makoto Abe, Shigeru Tamogami

**Affiliations:** Department of Biological Production, Akita Prefectural University, Akita 010-0195, Japan; Email: abeman@akita-pu.ac.jp (M.A.); tamo-chem@akita-pu.ac.jp (S.T.)

**Keywords:** *Fallopia sachalinensis*, herbivore-induced volatiles, methyl jasmonate, phenylacetonitrile, *Popillia japonica*

## Abstract

Phenylacetonitrile, (*E*)-β-ocimene, linalool, (*E*)-4,8-dimethyl-1,3,7-nonatriene and (*E*,*E*)-α-farnesene were identified as Japanese beetle, *Popillia japonica*, feeding-induced volatiles from the leaves of the giant knotweed, *Fallopia sachalinensis*, but not by mechanical damage. Volatile emission was also induced by treatment with a cellular signaling molecule, methyl jasmonate. These results suggest that volatiles will be synthesized *de novo* by a biotic elicitor from *P*. *japonica* oral secretion.

## 1. Introduction

Plants emit a series of characteristic volatile blends, such as terpenes and green leaf volatiles, when they are damaged by insect feeding. These herbivore-induced plant volatiles are known to either attract natural enemies of the herbivores or induce defense responses of other plants in the vicinity, and thus volatiles play an important role in plant defense against herbivores [1,2,3]. Plant volatile emissions are activated by not only elicitors found in herbivore oral secretions [4,5,6,7], but also treatment with a phytohormone, jasmonic acid (JA) [8], and its related compound, methyl jasmonate (MeJA) [3,9,10]. It has been shown that JA signaling lies downstream of insect feeding [11] and that exogenous MeJA is converted into jasmonoyl isoleucine (JA-Ile) via JA *in planta*, and the resulting active endogenous JA-Ile activates plant indirect defenses [12].

The Japanese beetle, *Popillia japonica* (Coleoptera: Scarabaeidae), is known to be a destructive pest in North America that damages leaves, flowers or fruits of more than 300 plant species [13]. Both female and male beetles are attracted by floral or fruit-like odors, such as geraniol, eugenol and phenethyl propionate, which enhance the attraction of males when treated with a sex pheromone, japonilure [14,15]. During our field research around Akita City in Japan, the beetles are often found on the leaves of the giant knotweed, *Fallopia sachalinensis* (Polygonaceae), and infested plants in the field smell like sweet grapes (our personal observation). The same grape-like odor is reproduced under laboratory conditions where the beetles are fed on *F*. *sachalinensis* leaves. In this context, we speculate that the odor will function for beetle attraction, and plan to investigate the volatile composition. Here we report the identification of volatiles from the leaves of *F*. *sachalinensis* infested by *P*. *japonica* and that an uncommon type of herbivore-induced volatile, phenylacetonitrile (benzyl cyanide), from *F*. *sachalinensis* is also induced by treatment with exogenous airborne MeJA.

## 2. Results and Discussion

**Figure 1:**
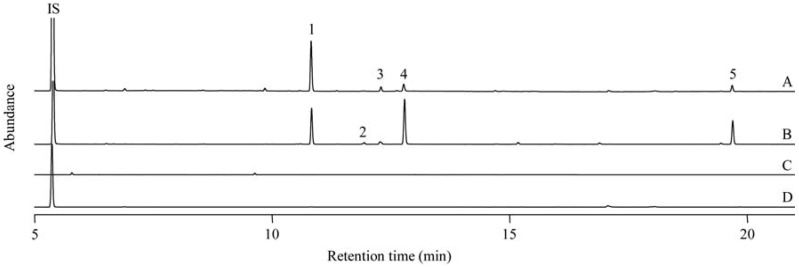
Typical gas chromatograms of volatiles from leaves of *F*. *sachalinensis* with different treatments. A, infested by *P*. *japonica*; B, treated with exogenous airborne MeJA; C, mechanically damaged; D, undamaged. The numbers of peaks represent 1, (*E*)-β-ocimene; 2, linalool; 3, DMNT; 4, phenylacetonitrile; and 5, (*E*,*E*)-α-farnesene.

The volatiles induced by *P*. *japonica* feeding were analyzed by GC–MS and the volatile components were identified as phenylacetonitrile, (*E*)-β-ocimene, linalool, DMNT and (*E*,*E*)-α-farnesene, as shown in Figure 1 and Table 1. Volatile emission was not observed from either undamaged leaves or mechanically damaged leaves. These results suggest that volatile emission will be induced by a herbivore-specific factor, an elicitor present in the oral secretion of *P*. *japonica*. It is known that a fatty acid-amino acid conjugate, volicitin, is a strong elicitor that is widely distributed in lepidopteran caterpillars [16] and also found in cricket and fruit fly [17]. Volicitin induces the emission of volatile blends composed of terpenes, green leaf volatiles and indole [4]. Inseptin is a peptidic elicitor found in oral secretions of lepidopteran larvae, *Spodoptera frugiperda*, which promote either defense-related phytohormone production or herbivore-induced volatile emission, such as DMNT [7]. Caeliferins have been identified as nonlepidopteran elicitors from the American bird grasshopper, *Schistocerca americana*, which also induce volatiles from plants similar to caterpillar feeding [5]; however, there is no example in which the elicitor was identified from Coleoptera and that the elicitor induces nitrile emission from plants. 

**Table 1:** Volatile composition from the leaf of *F*. *sachalinensis* with different treatments.

Compound	Composition (%) *^a^*			
	Infested by *P*. *japonica*	Treated with airborne MeJA	Mechanically damaged	Undamaged
( *E*)-β-Ocimene	71.3	33.0	n.d. *^b^*	n.d.
Linalool	0.6	1.6	n.d.	n.d.
DMNT	7.9	2.2	n.d.	n.d.
Phenylacetonitrile	10.3	39.9	n.d.	n.d.
( *E*,*E*)-α-Farnesene	9.8	23.3	n.d.	n.d.

*^a^* Percentages are based on GC peak area; *^b^* n.d. = not detected.

Four terpenes, (*E*)-β-ocimene, linalool, DMNT and (*E*,*E*)-α-farnesene, have been found as *P*. *japonica* feeding-induced volatiles from the leaves of grape, *Vitis labrusca* and crabapple, *Malus* spp. [18,19]; however, *F*. *sachalinensis* is an unique species that emits phenylacetonitrile infested by the same herbivore, *P*. *japonica*. Although it has not been determined whether these terpenes attract beetles, the beetles prefer the leaves of *V*. *labrusca* and *Malus* spp. infested by *P*. *japonica* feeding than undamaged leaves [18,19] and thus, the herbivore-induced volatiles are likely to serve as host location cues for the beetles. The above terpenes are well-known volatiles induced by insect feeding, while there are only a few examples in which phenylacetonitrile was found as an herbivore-induced volatile. Phenylacetonitrile has been found from insect-infested Manchurian ash, *Fraxinus mandshurica* [10] and *Brassica* spp. [8,20], and mite-infested apple leaves [21]. Phenylacetonitrile found from caterpillar-infested *B*. *rapa* has been shown to attract parasitoid wasps [20]. Phenylacetonitrile is also known as one of the floral scent components of Orchidaceae, such as *Diaphananthe pellucida* [22] and Cactaceae [23]. This volatile compound may be a potential chemical cue to attract various types of insects, for example, natural enemies, flower-visiting insects, and herbivores. 

**Figure 2:**
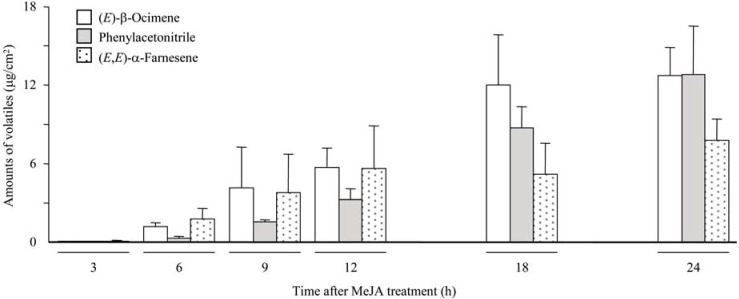
Time-dependent volatile emission from leaves of *F*. *sachalinensis* treated with airborne MeJA.

Herbivore-induced volatiles were qualitatively mimicked by exogenous airborne MeJA (Figure 1). Induction of the emission of phenylacetonitrile, (*E*)-β-ocimene and (*E*,*E*)-α-farnesene by MeJA was much higher than by insect feeding. The emission of the three major volatile components started 3 h after MeJA treatment. The emission of two terpenes increased and reached almost maximum 18 h after MeJA treatment. The increase of phenylacetonitrile emission, which initially lagged behind those of terpenes, was time dependent during the experimental period (24 h, Figure 2). All of the volatile emission triggered by MeJA was suppressed under constant dark conditions to 29–39% of that under constant light conditions (data not shown). It is suggested that light is essential for volatile production and/or emission together with activation of the JA signaling pathway. As *P*. *japonica* diurnally infests the leaves of *F*. *sachalinensis*, it is reasonable for *F*. *sachalinensis* to response under light conditions. It could also be favorable for *P*. *japonica* to use photoperiodic volatile emission from *F*. *sachalinensis* as an attractant. 

Plant nitriles are known as one of the degradation products of glucosinolates, mostly found in the Brassicaceae family. In this system, glucosinolates are initially hydrolyzed by myrosinase which catalyzes the glucosinolates when plant tissue is physically damaged by cutting, grinding or chewing [24]. In *F*. *sachalinensis*, nitrile emission was not observed when the plant tissue was mechanically damaged, suggesting that the nitrile was not derived from a corresponding glucosinolate. The induction of phenylacetonitrile emission from undamaged leaves by MeJA also indicates that the nitrile is synthesized *de novo* in *F*. *sachalinensis*. In microorganisms, nitrile is synthesized from the corresponding aldoxime, called aldoxime–nitrile pathway [25,26]. This could be one of the possible pathways to produce nitrile in *F*. *sachalinensis*. Phenylacetonitrile is likely to be derived from phenylalanine in plants through decarboxylation [23,27]. Phenylalanine is an important amino acid precursor yielding intermediates for functional metabolites. MeJA has been reported to induce the expression of phenylalanine ammonia lyase (PAL), which is involved in plant chemical defense [28,29]. PAL catalyzes the deamination of phenylalanine, and this process is quite different from that of the decarboxylation of phenylalanine, presumably led to phenylacetonitrile. The presence of phenylacetonitrile indicates that the decarboxylation pathway is preferably activated in *F*. *sachalinensis* by either insect feeding or exogenous MeJA.

## 3. Experimental Section

### 3.1. Plants and insects

Roots of the giant knotweed, *F*. *sachalinensis*, were collected at Kamishinjyo, Akita City, Japan in August, 2009. The roots were kept at room temperature in commercially available culture soil. New shoots were grown from the roots and then mature green leaves of *F*. *sachalinensis* originating from different roots were used for volatile collection. Japanese beetles, *P*. *japonica*, were collected on the leaves of *F*. *sachalinensis* in the same area as the collection site of *F*. *sachalinensis* in August, 2009 and 2010. They were reared on *F*. *sachalinensis* leaves in our lab for the period of the experiment. 

### 3.2. Plant volatile collection

Volatiles were collected from *F*. *sachalinensis* leaves infested by *P*. *japonica* (N = 6). Undamaged leaves were used as a negative control (N = 3). To identify the herbivore-induced volatiles from *F*. *sachalinensis* leaves, a mature leaf excised from the base of its petiole was placed in a glass container (1 L), and then three *P*. *japonica* beetles were introduced onto the leaf. The container was placed in a chamber at 25 °C under continuous light. Fifty microliters of acetone containing 20 μg/μL *n*-octane as an internal standard was added to the container after 24 h and then the volatiles in the container were collected using a solid phase micro extraction (SPME) fiber (65 μm Stable Flex PDMS/DVB, Supelco, PA, USA) for 30 min. The SPME fiber was then injected into the GC or GC–MS for 5min and the volatile components were analyzed by GC or GC–MS. 

To determine whether the volatiles were induced specifically by insect feeding, a mature leaf was bored using a pipet tip and then the volatiles from the mechanically damaged leaf were analyzed by the same procedure (N = 3). A mature leaf was also enclosed with a paper disk containing 2 μL MeJA without any contact and then the volatiles induced by exogenous airborne MeJA were analyzed by the same procedure as described above (N = 10). 

### 3.3. Chemical analyses

GC/MS analysis was carried out using a PerkinElmer Turbo Mass (Shelton, CT, USA) operated at 70 eV using a DB-5MS capillary column (Agilent Technologies, 30 m × 0.25 mm i.d., 0.25 μm film thickness) with helium career gas at 1.0 ml/min. The oven temperature was programmed to change from 50 °C (3 min holding) to 200 °C at 10 °C/min, followed by a 3 min hold and then the temperature was increased to 300 °C at 20 °C/min and held for 4 min. Both the injector temperature and the detector temperature were maintained at 250 °C. Phenylacetonitrile and linalool were identified by comparing their GC retention times and the mass spectra of authentic standards. (*E*)-β-Ocimene, (*E*)-4,8-dimethyl-1,3,7-nonatriene (DMNT) and (*E*,*E*)-α-farnesene were identified by the NIST MS library and according to previous results [12]. Quantification analysis of time-dependent changes of the volatiles from *F*. *sachalinensis* was performed using a Shimadzu GC-2010 (Kyoto, Japan) with a flame ionization detector under the same analytical conditions as GC/MS analysis. The volatiles were collected 3–24 h after treatment with MeJA and the amount of volatile emission per square of leaf was determined by the relative ratio of the peak area to that of the internal standard (each n = 5). 

## 4. Conclusions

Volatiles from the leaves of *F*. *sachalinensis* infested by *P*. *japonica* were determined by GC–MS. Phenylacetonitrile, an unique compound of herbivore-induced volatile, was found together with four terpenes, (*E*)-β-ocimene, linalool, DMNT and (*E*,*E*)-α-farnesene, which are well-known inducible volatiles by herbivore attack. Volatile emission was qualitatively mimicked by treatment with an exogenous airborne MeJA, suggesting that the synthesis of these volatiles was regulated by the JA signaling pathway. 

## References

[1] Turlings T.C., Tumlinson J.H., Lewis W.J. (1990). Exploitation of herbivore-induced plant odors by host-seeking parasitic wasps. Science.

[2] Paré P.W., Tumlinson J.H. (1999). Plant volatiles as a defense against insect herbivores. Plant Physiol..

[3] Kessler A., Baldwin I.T. (2001). Defensive function of herbivore-induced plant volatile emissions in nature. Science.

[4] Alborn H.T., Turlings T.C.J., Jones T.H., Stenhagen G., Loughrin J.H., Tumlinson J.H. (1997). An elicitor of plant volatiles from beet armyworm oral secretion. Science.

[5] Alborn H.T., Hansen T.V., Jones T.H., Bennett D.C., Tumlinson J.H., Schmelz E.A., Teal P.E.A. (2007). Disulfooxy fatty acids from the American bird grasshopper *Schistocerca americana*, elicitors of plant volatiles. Proc. Natl. Acad. Sci. USA.

[6] Mori N., Yoshinaga N., Sawada Y., Fukui M., Shimoda M., Fujisaki K., Nishida R., Kuwahara Y. (2003). Identification of volicitin-related compounds from the regurgitant of lepidopteran caterpillars. Biosci. Biotechnol. Biochem..

[7] Schmelz E.A., Carroll M.J., LeClere S., Phipps S.M., Meredith J., Chourey P.S., Alborn H.T., Teal P.E.A. (2006). Fragments of ATP synthase mediate plant perception of insect attack. Proc. Natl. Acad. Sci. USA.

[8] Bruinsma M., Posthumus M.A., Mumm R., Mueller M.J., van Loon J.J.A., Dicke M. (2009). Jasmonic acid-induced volatiles of *Brassica oleracea* attract parasitoids: Effects of time and dose, and comparison with induction by herbivores. J. Exp. Bot..

[9] Rodriguez-Saona C., Crafts-Brandner S.J., Paré P.W., Henneberry T.J. (2001). Exogenous methyl jasmonate induces volatile emissions in cotton plants. J. Chem. Ecol..

[10] Rodriguez-Saona C., Poland T.M., Miller J.R., Stelinski L.L., Grant G.G., de Groot P., Buchan L., MacDonald L. (2006). Behavioral and electrophysiological responses of the emerald ash borer, *Agrilus planipennis*, to induced volatiles of Manchurian ash, *Fraxinus mandshurica*. Chemoecology.

[11] Schmelz E.A., Alborn H.T., Banchio E., Tumlinson J.H. (2003). Quantitative relationships between induced jasmonic acid levels and volatile emission in *Zea mays* during *Spodoptera exigua* herbivory. Planta.

[12] Tamogami S., Rakwal R., Agrawal G.K. (2008). Interplant communication: Airborne methyl jasmonate is essentially converted into JA and JA-Ile activating jasmonate signaling pathway and VOCs emission. Biochem. Biophys. Res. Commun..

[13] Potter D.A., Held D.W. (2002). Biology and management of the Japanese beetle. Ann. Rev. Entomol..

[14] Ladd T.L., McGovern T.P. (1980). Japanese beetle: A superior attractant, phenethyl propionate + eugenol + geraniol, 3:7:3. J. Econ. Entomol..

[15] Klein M.G., Tumlinson J.H., Ladd T.L., Doolittle R.E. (1981). Japanese beetle (Coleoptera: Scarabaeidae): Response to synthetic sex attractant plus phenethyl propionate: eugenol. J. Chem. Ecol..

[16] Yoshinaga N., Alborn H.T., Nakanishi T., Suckling D.M., Nishida R., Tumlinson J.H., Mori N. (2010). Fatty acid-amino acid conjugates diversification in lepidopteran caterpillars. J. Chem. Ecol..

[17] Yoshinaga N., Aboshi T., Ishikawa C., Fukui M., Shimoda M., Nishida R., Lait C.G., Tumlinson J.H., Mori N. (2007). Fatty acid amides, previously identified in caterpillars, found in the cricket *Teleogryllus taiwanemma* and fruit fly *Dorosophila melanogaster* larvae. J. Chem. Ecol..

[18] Loughrin J.H., Potter D.A., Hamilton-Kemp T.R. (1995). Volatile compounds induced by herbivory act as aggregation kairomones for the Japanese beetle (*Popillia japonica* Newman). J. Chem. Ecol..

[19] Loughrin J.H., Potter D.A., Hamilton-Kemp T.R., Byers M.E. (1996). Role of feeding-induced plant volatiles in aggregative behavior of the Japanese beetle (Coleoptera: Scarabaeidae). Environ. Entomol..

[20] Kugimiya S., Shimoda T., Tabata J., Takabayashi J. (2010). Present or past herbivory: A screening of volatiles released from *Brassica rapa* under caterpillar attacks as attractants for the solitary parasitoid, *Cotesia vestails*. J. Chem. Ecol..

[21] Takabayashi J., Dicke M., Posthumus M.A. (1994). Volatile herbivore-induced terpenoids in plant-mite interactions: Variation caused by biotic and abiotic factors. J. Chem. Ecol..

[22] Kaiser R. (1993). The Scent of Orchids: Olfactory and Chemical Investigations.

[23] Kaiser R., Tollsten L. (1995). An introduction to the scent of cacti. Flavour Frag. J..

[24] Lambrix V., Reichelt M., Mitchell-Olds T., Kliebenstein D.J., Gershenzon J. (2001). The *Arabidopsis* epithiospecifier protein promotes the hydrolysis of glucosinolates to nitriles and influences *Trichoplusia ni* herbivory. J. Plant Cell.

[25] Asano Y., Kato Y. (1998). *Z*-Phenylacetaldoxime degradation by a novel aldoxime dehydratase from *Bacillus* sp. strain OxB-1. FEMS Microbiol. Lett..

[26] Kato Y., Tsuda T., Asano Y. (2007). Purification and partial characterization of *N*-hydroxy-L-phenylalanine decarboxylase/oxidase from *Bacillus* sp. strain OxB-1, an enzyme involved in aldoxime biosynthesis in the “aldoxime–nitrile pathway”. Biochim. Biophys. Acta.

[27] Dewick P.M. (1997). Medicinal Natural Products—A Biosynthetic Approach-.

[28] Koukol J., Conn E.E. (1961). The metabolism of aromatic compounds in higher plants. J. Biol. Chem..

[29] Gundlach H., Muller M.J., Kutchan T.M., Zenk M.H. (1992). Jasmonic acid is a signal transducer in elicitor-induced plant cell cultures. Proc. Natl. Acad. Sci. USA.

